# Adolescent mothers, self-care and childcare: content validation of an Event History Calendar

**DOI:** 10.1590/1980-220X-REEUSP-2022-0314en

**Published:** 2023-03-31

**Authors:** Jaqueline Silva Santos, Sarah Neill, Débora Falleiros de Mello

**Affiliations:** 1 Secretaria de Estado de Saúde de Minas Gerais, Superintendência Regional de Saúde de Passos, Passos, MG, Brazil.; 2 University of Plymouth, Faculty of Health, School of Nursing and Midwifery, Plymouth, United Kingdom.; 3 Universidade de São Paulo, Escola de Enfermagem de Ribeirão Preto, Departamento de Enfermagem Materno-Infantil e Saúde Pública, Ribeirão Preto, SP, Brazil.

**Keywords:** Child Healt, Adolescent Healt, Primary Health Car, Nursin, Validation Stud, Salud Infantil, Salud del Adolescente, Atención Primaria de Salud, Enfermería, Estudio de Validación, Saúde da Criança, Saúde do Adolescente, Atenção Primária à Saúde, Enfermagem, Estudo de Validação

## Abstract

**Objective:**

To validate the content of the tool Event History Calendar Adolescent Mother: strengthening self-care and child care.

**Method:**

Methodological study using the Delphi technique, conducted in two rounds, involving 37 nursing specialists. In data collection, from December/2019 to August/2020, a semi-structured questionnaire composed of 47 items related to the two dimensions of the tool: Self-care and Child Care was used. The Content Validity Index ≥ 0.80 was used to assess agreement among the experts. Qualitative elements were analyzed for clarity and comprehensiveness of content.

**Results:**

In the first round, 46 items showed Content Validity Index ≥ 0.80. The qualitative elements pointed out more clarity for the adolescent audience. After the changes, the tool presented 30 items. In the second round, the 30 items evaluated achieved Content Validity Index ≥ 0.80. The qualitative considerations were translated into modifications in the content and sequence in the final version of the tool.

**Conclusion:**

The validated tool obtained adequate evaluation of the items of each dimension, related to adolescent mother self-care and child care, with a high degree of comprehensibility.

## RESUMO

**Objetivo:**

Validar o conteúdo da ferramenta *Event History Calendar* Mãe Adolescente: fortalecendo o autocuidado e o cuidado da criança.

**Método:**

Estudo metodológico com a técnica Delphi, realizado em duas rodadas, envolvendo 37 especialistas de enfermagem. Na coleta de dados, de dezembro/2019 a agosto/2020, foi utilizado um questionário semiestruturado composto por 47 itens relacionados às duas dimensões da ferramenta: Autocuidado e Cuidado da criança. O Índice de Validade de Conteúdo ≥ 0,80 foi utilizado para avaliar a concordância entre os especialistas. Elementos qualitativos foram analisados quanto à clareza e abrangência do conteúdo.

**Resultados:**

Na primeira rodada, 46 itens apresentaram Índice de Validade de Conteúdo ≥ 0,80. Os elementos qualitativos apontaram necessidade de maior clareza para o público adolescente. Após as alterações, a ferramenta apresentou 30 itens. Na segunda rodada, os 30 itens avaliados alcançaram Índice de Validade de Conteúdo ≥ 0,80. As considerações qualitativas foram traduzidas em modificações no conteúdo e sequência na versão final da ferramenta.

**Conclusão:**

A ferramenta validada obteve avaliação adequada dos itens de cada dimensão, relacionados ao autocuidado da mãe adolescente e cuidado da criança, com alto grau de compreensibilidade.

## RESUMEN

**Objetivo:**

Validar el contenido de la herramienta *Event History Calendar* Madre Adolescente: fortaleciendo el autocuidado y el cuidado de los hijos.

**Método:**

Estudio metodológico mediante la técnica Delphi, realizado en dos rondas, en el que participaron 37 expertos en enfermería. En la recopilación de datos, de diciembre de 2019 a agosto de 2020, se utilizó un cuestionario semiestructurado compuesto por 47 ítems relacionados con dos dimensiones de la herramienta: Autocuidado y Cuidado del niño. Se utilizó el Índice de Validez del Contenido ≥ 0,80 para evaluar el acuerdo entre los expertos. Se analizaron los elementos cualitativos para comprobar la claridad y exhaustividad del contenido.

**Resultados:**

En la primera ronda, 46 ítems presentaron Índice de Validez de Contenido ≥ 0,80. Los elementos cualitativos apuntan a la necesidad de una mayor claridad para el público adolescente. Tras los cambios, la herramienta presentaba 30 ítems. En la segunda ronda, los 30 ítems evaluados alcanzaron un Índice de Validez de Contenido ≥ 0,80. Las consideraciones cualitativas se tradujeron en modificaciones del contenido y la secuencia en la versión final de la herramienta.

**Conclusión:**

El instrumento validado obtuvo evaluación adecuada de los ítems de cada dimensión, relacionados al autocuidado de la madre adolescente y al cuidado del niño, con alto grado de comprensibilidad.

## INTRODUCTION

When dealing with adolescent health care, it is important to consider the multiplicity of aspects that influence the adolescence process, such as those related to family, friends, school context, search for rights and plans for the future^(1)^, and the need for timely identification of possible vulnerabilities experienced in this life period^(2)^.

Regarding the care of adolescents, interventions are fundamental, mainly related to their reproductive and sexual health, violence prevention and attention to mental health, involving multisectoral actions, which have been discussed in accordance with the Sustainable Development Goals^(3)^. The chances of favorable outcomes and of reaching potentialities in the adolescence and adulthood phases are also linked to early childhood interventions, strengthening longitudinal follow-up for healthy growth and development and safe parenting practices, which provide a solid foundation^(4,5)^.

Adolescent motherhood may involve the experience of changes related to the reduction of time for oneself, the adaptation to new responsibilities and the construction of the maternal role, as well as the need to reconcile childcare and study, which requires support, routine organization, and realignment of life projects^(6)^. Thus, depending on the context experienced, adolescent mothers may experience different feelings and emotions^(6)^, such as concerns, fears, and loneliness^(7)^, but also joy, satisfaction, and confidence^(6)^.

In terms of effective care to health of adolescent mothers, the achievement of maternal mortality goals and the improvement in the coverage of health services are pointed out as essential, paying attention to inequalities and vulnerabilities between adolescent (15–19 years old) and adult (20–34 years old) mothers in prenatal care, birth, and puerperium^(8)^. The knowledge and proximity to the life settings of adolescent mothers allow health professionals to offer more support in the provision of care^(6,9–12)^. For these reasons, it is essential that health services are organized to receive, monitor and intervene with adolescent mothers^(11)^, highlighting the potentialities of the work of nurses in the context of primary health care (PHC)^(1,13)^.

Based on the recognition of the singularities of the phenomenon of maternity in adolescence, the use of tools that enable the expression of the needs of adolescent mothers and the contributions to their life contexts becomes relevant^(6,12,14)^. The Event History Calendar (EHC) tool contains a structure with mechanisms for recording autobiographical memory, data collection, and retrospective reports on different dimensions of a person’s life^(15,16,17,18)^.

A study^(15)^ presented information on elements of the EHC (structure, functioning and applicability in nursing research with adolescents), and research based on the analysis of experiences and situations lived by Brazilian adolescent mothers between 12 and 18 years^(6)^, provided inputs for the development of the “EHC – Adolescent Mother: strengthening self-care and child care” (EHCAM), structured in two dimensions: Self-care and Child Care.

The dimensions of the EHCAM tool allow monitoring aspects related to the life and health of adolescent mothers, child care practices, and the presence or absence of a support network. The dimension Adolescent mother’s self-care presents items that aim to identify care needs and factors that protect or make her health and well-being vulnerable, in order to make nursing care consistent with her specificities. The dimension Child Care seeks to identify the child’s care needs and the insecure and vulnerable aspects that may hinder the promotion, prevention, and protection of the child’s health, as well as items related to the adolescent mother’s previous experiences, paternal participation, child growth and development, and routine activities. Thus, the items that make up the EHCAM tool can provide inputs for comprehensive, singular, and safe care, constituting potential bases for good practices developed by nurses.

The understanding of the need for welcoming and dialogical approaches that promote reflection, construction of knowledge and skills for self-care and care of the child^(13)^, the configuration of an EHC as an appropriate tool for the care of adolescents^(1,6,15,16)^ and the importance of validating the content of an instrument^(19,20)^ motivated the present research. Thus, the aim of the study is to validate the content of the EHCAM tool, considering its potential applicability in the clinical practice of PHC nurses.

## METHODS

### Study Design

Methodological study of content validation of the EHCAM tool, guided by the SQUIRE 2.0 guidelines of the EQUATOR network. The Delphi technique was used with a group of expert participants, characterized by the identification of consensus and disagreement on the topic, interconnectedness, and potential for knowledge integration^(21)^.

### Setting

The research was conducted in Brazil, in a remote format, with participants from the South, Southeast, Center-West, Northeast and North regions.

### Population, Selection Criteria and Sample

The study population was constituted by the selection of researchers and nursing specialists, identified by the curriculum in virtual format available in the Lattes Curriculum Platform of the National Council for Scientific and Technological Development (CNPq-Brazil).

Inclusion criteria were: nurse, with a doctoral degree in health care, academic and/or professional experience in adolescent and/or child health. Duplicate entries were excluded. In the period December 2019 to January 2020, a list was compiled with the names and electronic mail (email) contacts of 179 experts who met the inclusion criteria. Those who did not respond to the survey invitation email were excluded. Thirty-seven experts participated in the study.

### Data Collection

The study was conducted from December 2019 to August 2020. Initially, an email was sent to each selected specialist containing: i) invitation letter with the purpose of the study and explanations about the EHCAM tool; ii) Informed Consent Form (ICF); and iii) first version of the questionnaire with instructions.

In the Self-Care dimension of the EHCAM tool, the items refer to maternal age, education, occupation, daily activities/time use, relationships, lifestyle, feelings/concerns, prejudice/discrimination for being a teen mother, women’s health care, and plans for the future. In the dimension Child Care, the items refer to sources of care, child routine, child growth and development, preventive actions and health promotion.

In the first round of the Delphi technique, between February 26 and June 11, 2020, a semi-structured questionnaire composed of 47 items was used, making up the two dimensions of the EHCAM tool. The Self-Care dimension contains 24 items and the Child Care dimension 23 items. For each item, there is a numerical scale from one to four (1- not very relevant; 2- partially relevant; 3- relevant; 4- extremely relevant) and an open space for writing comments and/or suggestions from experts. At the end of each dimension, there is another open space to respond as to the clarity and scope of the content. A deadline of 20 days was set for responding with the signed ICF and sending the completed questionnaire. After receiving the 37 completed questionnaires, the data referring to the two dimensions of the tool were compiled and analyzed.

In the second round of the Delphi technique, between June 12, 2020 and August 19, 2020, an email was sent individually to the 37 study participants containing the second version of the questionnaire with instructions and the experts’ considerations about the first round. A semi-structured questionnaire consisting of 30 items, 15 items for each of the two dimensions, was used for the experts’ reassessment. This questionnaire contained the initial description of the item, the Content Validity Index (CVI) obtained by the item in the first round, the reformulated item, and the numeric scale with values from 1 to 4 for the evaluation of the reformulated item by the experts. At the end of each dimension, a space was inserted for comments from the experts. A deadline of 20 days was set for a response. Individual e-mails were sent to the specialists with reminders about the deadline. In this round, 24 specialists participated (64.9%) of the group participating in the first round.

### Analysis and Treatment of Data

In the data analysis, the CVI was used to evaluate the agreement between the experts^(22)^, applied to each item evaluated. The overall CVI was used to assess the agreement in the dimension as a whole, corresponding to the average of the CVI values of each related item^(22)^. The CVI for each item was calculated by adding the number of answers 3 or 4 divided by the total number of valid answers received. The overall CVI of the dimension was calculated by adding the CVI of each item, divided by the total number of items in the dimension. The minimum value of CVI ≥ 0.80 was established for the evaluation of each item and for the overall evaluation of the dimension.

In order to conduct the analysis process, the specialists’ comments and suggestions were also considered, aiming to include qualitative elements of each dimension, particularly regarding the clarity and comprehensiveness of the content. The qualitative answers were compiled and organized in individual files for each item. The consensus of the group of specialists was obtained through two rounds of the Delphi technique.

### Ethical Aspects

The study followed the norms and guidelines of Resolution No. 466/2012, being analyzed and cleared by the Research Ethics Committee, opinion No. 3,627,170, in the year 2019. An ICF was used, signed by the participants of this research. To ensure anonymity, in the first round of the Delphi technique, participants were named E1, E2…E37. In the second round, they were referred to as E1, E2…E24.

## RESULTS

### Characteristics of the Expert Participants

The profile of the 37 participants presents an age range from 30 to 66 years, female (94.6%), graduation in nursing between 8 and 43 years, and most (81.1%) with accumulated experience in adolescent and/or child health. Regarding education, 25 participants (67.6%) had doctoral degrees, seven had postdoctoral degrees (18.9%), four (10.8%) were full professors, and one (2.7%) was a full professor.

The participants worked in different Brazilian regions, 19 (51.3%) in the Southeast, nine (24.3%) in the Northeast, four (10.8%) in the South, four (10.8%) in the Midwest, and one (2.7%) in the North. Regarding the place of work, 31 participants (83.8%) worked in public universities, four (10.8%) in private universities and two (5.4%) linked to PHC services.

### The First and Second Round by Delphi Technique

In the first round of the Delphi technique, the dimensions of the EHCAM tool were evaluated by 37 experts. In the Self-Care dimension, the 24 items presented CVI ≥ 0.80 and the overall CVI of the dimension was 0.95. In the Child Care dimension, 22 items showed CVI ≥ 0.80, the item regarding solidarity had a CVI equal to 0.67, and the overall CVI of the dimension was 0.96.

In the first round, the qualitative elements coinciding with the content of the dimensions were predominantly focused on the need for greater clarity for the adolescent audience. This example expresses this aspect: *I think teenagers will have difficulty in understanding the presentation topic, I suggest presenting it in a question format and, when possible, direct it to what is intended to be known, in order to contribute to the care provided to mother and child* (E6).

Some items were considered by the specialists to have very similar contents: *There is very similar previous question about the school* (E7); *In the employment item I considered there was a repetition* (E32). The notes were unified in questions to cover different aspects and avoid repetition. Other items were expanded, with the inclusion of specific questions that contemplated aspects indicated by the specialists, as in these examples: *It is important to have a sub-item on the body perception and satisfaction with the adolescent’s body image* (E8); *Inclusion of an item referring to illnesses/morbidities and the care with home medications and/or self-medication of the child* (E16).

There were suggestions of insertions of items, one for the dimension Self-Care: *In the item on relationships, I suggest, if you think it is pertinent, to add the relationship with health professionals of the services she used. It could shed light on the weaknesses and strengths of health services from the adolescent’s perspective* (E17), and one in the dimension Child Care: *In the item Child Care Sources, I would include a sub-item related to the father’s participation* (E3).

In the analysis of the specialists’ comments and suggestions, those changes concerning the objective of the study were included, with items modified for specific and exemplified questions, seeking greater clarity and adequacy, exclusion of items to avoid repetition, and inclusion of items to include other aspects. As of the first round, the Self-Care dimension contained 15 items and the Child Care dimension 15 items.

In the second round, the EHCAM dimensions were evaluated by 24 experts. In the Self-Care dimension, the 15 items had CVI ≥ 0.80 and the overall CVI was 0.95. In the Child Care dimension, the 15 items had CVI ≥ 0.80 and an overall CVI of the dimension of 0.96.

Regarding the qualitative elements of the dimensions, the experts suggested occasional modifications regarding the form of presentation and in some contents. In both dimensions, in the items composed of more than one question in sequence, each one was placed in a separate line, seeking greater clarity in the presentation and in obtaining answers.

With regard to contents, more specificity in the wording of questions was suggested: *‘Do you self-medicate?’, I suggest ‘Do you use medication without it having been indicated by your doctor?*’ (E22); *‘Do you know what to do when the child has colic, fever, eruption of teeth?’, replace eruptions by birth of teeth* (E22). The expansion of content was also pointed out: *‘How do you have fun?’, also question the religious aspect* (E10); *‘How do you take care of yourself?’, clarify what is meant by care for self-esteem* (E7); *‘What care do you take to protect your child*?’, *protecting the child is much more than fulfilling biomedical precepts or preventing accidents. The bond protects, even from infant malnutrition* (E11); *‘Does the father of the child participate in caregiving? what care(s) does he provide for the child?’, I recommend giving examples: preparing and offering food, bathing, changing diapers, washing and ironing clothes, playing, going for a walk, taking to the health services* (E8).

Other suggestions from the specialists were related to the greater detail and taking apart of items to give more value to the content in the questions: *‘Do you use any method to avoid pregnancy? If yes, which one?’, I suggest a separate item, providing a diagnosis of needs* (E10); *‘How do you see yourself?’ and ‘Are you satisfied with your body?’, one question is how I see myself and my body, even more in the adolescent segment, another is my objective conditions to take care of myself* (E11).

A change in the sequence of questions was also pointed out: *‘Is the child under the care of someone else (who?) or is he/she in daycare/school? If yes: at what time (e.g., morning, afternoon, all day)?’, I suggest moving this item to after growth and place it next to the other item on development/interaction* (E7).

**Chart 1:**
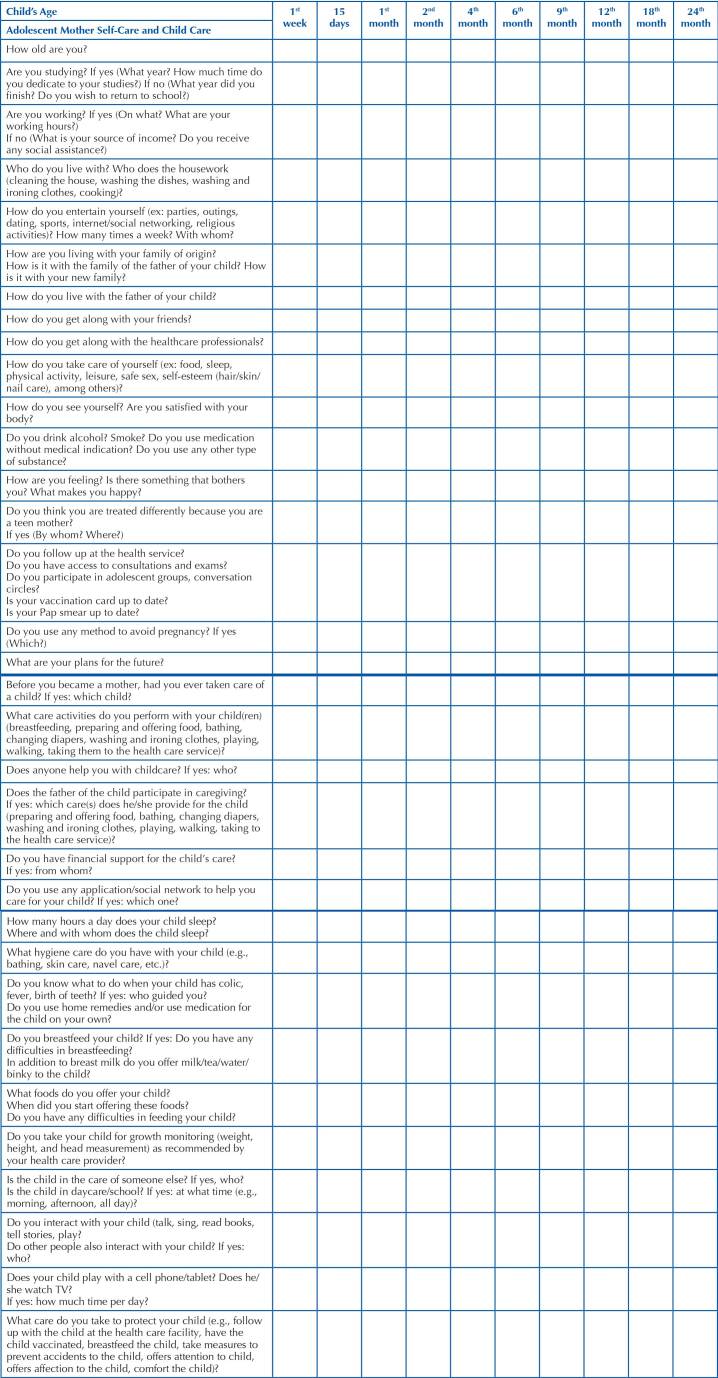
Version of the EHCAM with content validation by nursing specialists in adolescent and/or child health – Ribeirão Preto, SP, Brazil, 2020.

In view of the specialists’ comments, these questions were reformulated. The dimension Self-Care was composed of 17 items and the dimension Child Care of 15 items, resulting in the final version of the EHCAM tool, shown in Chart 1.

## DISCUSSION

The content validation process of the EHCAM tool was completed after two rounds with experts by the Delphi technique. In the second round, all items evaluated in both dimensions reached CVI ≥ 0.80. The overall CVI of the Self-Care dimension was 0.95 and of the Child Care dimension was 0.96. The qualitative elements were translated into the content and contributed to the final version of the tool.

The CVI is pointed out as relevant, and even when the Delphi rounds produce an overall CVI of 0.87, the experts suggest changes in the content, which can improve the final version^(23)^. The validated EHCAM tool, composed of 17 items in the Self-Care dimension and 15 items in the Child Care dimension, includes relevant aspects for comprehensive health care.

In adolescence, the singularities related to the experience of pregnancy, childbirth and daily care of the child point to multidimensional needs of the adolescent mother that must be considered in health care practices^(6)^. In this context, the elements that make up the Self-Care dimension in the EHCAM seek to help identify the profile of the adolescent mother and the protection and vulnerability factors present in her life context.

The events related to child care can be understood as daily learning for the teen mother, which signals the need to build strategies for coping with vulnerabilities and strengthening the support network^(6)^. In the dimension Child Care in the EHCAM, the elements are focused on different aspects of the care process related to knowledge, experience, skills and support network, with items that address the child’s biopsychosocial needs regarding hygiene, feeding, sleep, growth monitoring, stimulation of child development, child interaction and protection.

Such aspects focus on the importance of the context of adolescent mothers, for the understanding of circumstances and facing barriers, and for the construction of safe self-care and protective care with the child’s health^(1,6)^. In this context, the phenomenon of maternity in adolescence requires a unique nursing care for adolescent mothers, grounded in supportive practice, without judgments, with understanding of their life context and needs^(10)^ and the construction of autonomy^(6–11)^.

With regards to adolescent health care, in general, the EHC approach has the potential to support communication between nurses and adolescents^(1,15)^. In the present study, the items of the EHCAM dimensions seek to contribute to identify the care needs of the adolescent mother and child, through an approach that favors the interaction and communication between nurses and adolescents focusing on life events, including parenting. Thus, these particularities are pertinent to the practices of PHC nurses with adolescents in the context of parenthood^(13,24)^.

In the PHC field, the interventions with the parental caregivers are very important for the continuity of care, placing both the woman-child dyad^(25)^ and the perspectives of parental mutuality at the center of the care, for the connection and amplified understanding of the situations experienced^(26)^. Nurses are pointed out as the main source of support for adolescent mothers to identify stress and stressors, establish a guiding and reflective process, and strengthen the feeling of well-being through care, education, consultation, and health coordination interventions^(7)^.

On this path, nursing care for adolescent mothers gains emphasis when it creates meaningful spaces for listening, proximity, and bonding, favors the sharing of experiences, broadens the professional gaze on life trajectories and social and health determinants^(1)^, and considers the perspectives of the adolescent mother in the elaboration of autonomy and development^(24,27)^.

The EHCAM, despite presenting itself as an innovative structured tool, allows flexibility in the approach and fertile data collection, allowing to trigger the adolescent mothers’ own experiences and stimulating reflective processes about the lived situations, intrinsic to the conformation of an EHC^(15,16)^. The EHCs are referred to in the international scientific literature for use with adolescents in general^(14,16,28)^ and, in the present study, there was a central focus on adolescent mothers in the context of parenthood. They are mainly used by means of face-to-face or telephone interviews, with future potentiality of being self- administered by means of an Internet application^(28)^.

Some studies point out the need to listen to adolescent mothers so that they can share stories and experiences^(29)^, which allows the identification of individual contexts and circumstances and the understanding of the adolescents’ perspectives^(1,6)^. Home visit interventions performed by nurses with adolescents during pregnancy and the first two years of the child’s life have generated significant positive effects on maternal emotional and verbal responsiveness, infant expressive language development, and opportunities for variety in the child’s daily stimulation^(30)^. Thus, in the professional practice of nurses, it is important to build the care centered on the adolescent mother^(10)^, in communicative processes in health promotion practices in adolescence^(1,13)^, like the present EHCAM tool.

The limitations of the study are related to a reality of knowledge and experience of the participating specialists. The analysis obtained was satisfactory in order to answer the objective proposed in the present investigation. The appearance validation of the EHCAM tool will be done a posteriori. Moreover, the validation of the tool with adolescent mothers and nurses working in PHC services, specifying the ethnicity and insertion of different parental caregivers, will be relevant in future research.

## CONCLUSIONS

The EHCAM tool was validated, obtaining an adequate evaluation of the items of each dimension, related to the adolescent mother’s self-care and child care, with a high degree of comprehensibility.

In the context of communicative processes between adolescent mothers and nurses, the EHCAM tool may contribute to the improvement of nursing practice. It offers support for collecting life events and vulnerable situations, providing recognition of needs inherent to adolescence in the context of parenting. Consequently, the tool can illuminate the development of longitudinal care plans by nurses in PHC, considering the relational, educational and communicative dimensions of care and the elements pertinent to parenting for adolescents in the face of difficulties, events, learning and challenges in their lives.

## References

[1] Santos JS, Andrade RD, Silva MAI, Mello DF (2020). Nurse to adolescent health communication process: approach to Event History Calendar. Rev Bras Enferm..

[2] Ferguson J, Mathur S, Armstrong A (2021). Assessing the vulnerability and risks of adolescent girls and young women in East and Southern Africa: a preliminary review of the tools in use. Trop Med Infect Dis..

[3] George A, Jacobs T, Ved R, Jacobs T, Rasanathan K, Zaidi SA (2021). Adolescent health in the Sustainable Development Goal era: are we aligned for multisectoral action?. BMJ Glob Health..

[4] Jeong J, Franchett EE, Oliveira CVR, Rehmani K, Yousafzai AK (2021). Parenting interventions to promote early child development in the first three years of life: a global systematic review and meta-analysis. PLoS Med..

[5] Zhang L, Ssewanyana D, Martin MC, Lye S, Moran G, Abubakar A (2021). Supporting child development through parenting interventions in low- to middle-income countries: an updated systematic review. Front Public Health..

[6] Santos JS, Andrade RD, Silva MAI, Mello DF (2021). Strengthening self-care and child care of adolescent mothers through an event history calendar. J Pediatr Nurs..

[7] Tirgari B, Rayyani M, Cheraghi MA, Mangeli M (2020). Experiences of iranian teen mothers with parenting stress: a qualitative study. Compr Child Adolesc Nurs..

[8] Li Z, Patton G, Sabet F, Subramanian SV, Lu C (2020). Maternal healthcare coverage for first pregnancies in adolescent girls: a systematic comparison with adult mothers in household surveys across 105 countries, 2000–2019. BMJ Glob Health..

[9] Gurung R, Målqvist M, Hong Z, Poudel PG, Sunny AK, Sharma S (2020). The burden of adolescent motherhood and health consequences in Nepal. BMC Pregnancy Childbirth..

[10] Quosdorf A, Peterson WE, Rashotte J, Davies B (2020). Connecting with adolescent mothers: perspectives of hospital-based perinatal nurses. Glob Qual Nurs Res..

[11] Govender D, Taylor M, Naidoo S (2020). Adolescent pregnancy and parenting: perceptions of healthcare providers. J Multidiscip Healthc..

[12] Leal CCG, Gomes-Sponholz FA, Mamede FV, Silva MAI, Oliveira NTB, Leite AM (2018). *Photovoice*: method experiment research with adolescent mothers. Esc Anna Nery..

[13] Andrade RD, Hilário JSM, Santos JS, Silva JP, Fonseca LMM, Mello DF (2020). Maternal-child nursing care for adolescent mothers: health education. Rev Bras Enferm..

[14] Munro-Kramer ML, Fava NM, Banerjee T, Darling-Fisher CS, Pardee M, Villarruel AM (2017). The effect of a youth-centered Sexual Risk Event History Calendar (SREHC) assessment on sexual risk attitudes, intentions, and behavior. J Pediatr Health Care..

[15] Martyn KK, Belli RF (2002). Retrospective data collection using event history calendars. Nurs Res..

[16] Martyn KK, Saftner MA, Darling-Fisher CS, Schell MC (2013). Sexual risk assessment using event history calendars with male and female adolescents. J Pediatr Health Care..

[17] Axinn WG, Chardoul S, Gatny H, Ghimire DJ, Smoller JW, Zhang Y (2020). Using life history calendars to improve measurement of lifetime experience with mental disorders. Psychol Med..

[18] Saftner M, Thompson M, Ngabirano TD, McMorris BJ (2020). Adaptation of the event history calendar for Ugandan adolescents. Glob Health Promot..

[19] Fernández-Gómez E, Martín-Salvador A, Luque-Vara T, Sánchez-Ojeda MA, Navarro-Prado S, Enrique-Mirón C (2020). Content validation through expert judgement of an instrument on the nutritional knowledge, beliefs, and habits of pregnant women. Nutrients..

[20] Elangovan N, Sundaravel E (2021). Method of preparing a document for survey instrument validation by experts. MethodsX..

[21] Niederberger M, Spranger J (2020). Delphi technique in health sciences: a map. Front Public Health..

[22] Polit DF, Beck CT (2006). The content validity index: are you sure you know what’s being reported? critique and recommendations. Res Nurs Health..

[23] Lima ACMACC, Bezerra KC, Sousa DMN, Rocha JF, Oriá MOB (2017). Development and validation of a booklet for prevention of vertical HIV transmission. Acta Paul Enferm..

[24] Alarcão FSP, Shephard E, Fatori D, Amável R, Chiesa A, Fracolli L (2021). Promoting mother-infant relationships and underlying neural correlates: results from a randomized controlled trial of a home-visiting program for adolescent mothers in Brazil. Dev Sci..

[25] World Health Organization (2022). WHO recommendations on maternal and newborn care for a positive postnatal experience [Internet].

[26] Baldini PR, Lima BJ, Camilo BHN, Pina JC, Okido ACC (2021). Effect of parental mutuality on the quality of life of mothers of children with special health needs. Rev Lat Am Enferm..

[27] Bain LE (2021). Understanding the meaning of autonomy in adolescent pregnancy decision-making: lessons from Ghana. Pan Afr Med J..

[28] West BT, Axinn WG, Couper MP, Gatny H, Schroeder H (2022). A web-based event history calendar approach for measuring contraceptive use behavior. Field Methods..

[29] Pillay N (2021). ‘There is no more future for me? Like really, are you kidding?’: agency and decision-making in early motherhood in an urban area in Johannesburg, South Africa. Glob Health Action..

[30] Fatori D, Zuccolo PF, Shephard E, Brentani H, Matijasevich A, Ferraro AA (2021). A randomized controlled trial testing the efficacy of a nurse home visiting program for pregnant adolescents.. Sci Rep..

